# Defining competency in flexible cystoscopy: a novel approach using cumulative Sum analysis

**DOI:** 10.1186/s12894-016-0143-9

**Published:** 2016-06-13

**Authors:** Kenneth R. MacKenzie, Jonathan Aning

**Affiliations:** Department of Urology, The Newcastle upon Tyne Hospitals NHS Foundation Trust, Freeman Hospital, Newcastle-Upon-Tyne, NE7 7DN UK

**Keywords:** Flexible cystoscopy, Cumulative sum, CUSUM analysis, Learning curve

## Abstract

**Background:**

Flexible cystoscopy (FC) is one of the most frequently performed urological intervention. Cumulative sum analysis (CUSUM) allows objective assessment of a proceduralist’s performance to ensure acceptable outcomes. This study investigated the application of CUSUM to assess a trainee’s learning curve and maintenance of competence in performing FC.

**Methods:**

A single urology trainee, with no previous experience of FC, performed FCs between August 2013 and February 2014. For assessment FC was divided into 5 steps. Each step was assigned a CUSUM completion score. The primary outcome measure was successful performance of a complete FC. Prospective data were collected and analysed using CUSUM.

**Results:**

In total, 419 FCs were performed. Acceptable performance of FC was achieved by the 122^nd^ procedure. Complete assessment of the ureteric orifices and trigone was the most difficult step of FC to achieve consistently. Competence for complete FC was achieved following 289 procedures.

**Conclusion:**

CUSUM analysis objectively assesses acquisition of competence in flexible cystoscopy. Recommended indicative numbers may underestimate the number of FCs trainees require to achieve, and maintain, competency. Validation of CUSUM method in a larger cohort of trainees should be considered.

**Electronic supplementary material:**

The online version of this article (doi:10.1186/s12894-016-0143-9) contains supplementary material, which is available to authorized users.

## Background

Flexible cystoscopy (FC) is a vital diagnostic and therapeutic urological procedure, which enables immediate visual assessment of both the urethra and bladder.

FC comprises multiple steps, each of which require varying degrees of endoscopic skill. It is often assumed that competence in performing FC is achieved in the early years of urological training or within a limited number of procedures. Guidance informing assessment of competency in performing FC is limited. In 2000, a working party of the British Association of Urological Surgeons (BAUS) recommended that a minimum of 60 FCs should be performed under supervision to achieve technical competence [1]. This number has not been validated. It is now recognised that individuals training to perform a procedure, acquire skills at different rates [2]. Indicative numbers are a weak method of assessing competence and fail to identify or aid struggling trainees. There is a need for a more objective method to assess competency and guide training.

Cumulative Sum (CUSUM) analysis is a statistical tool that can be used to evaluate the development of competence in defined tasks [3]. CUSUM analysis has previously been used to chart learning curves and maintaining competency in surgical techniques, but not in FC, in vivo [3].

The aim of this study was to prospectively define the learning curve in FC of a surgical trainee with no previous FC experience and to evaluate the role of CUSUM as an objective measure of achieving and maintaining competency.

## Methods

### Setting

From August 2013 to February 2014 a Trust Grade in Urology, 2 years post qualification, with no previous experience in FC or endoscopy maintained a prospective database of all FCs performed at Weston General Hospital, North Somerset. The trainee intended to pursue a career in Urology and observed ten FCs prior to starting to perform FC. All FCs were performed using a flexible video cystoscope (Karl Storz, Germany). A senior Specialty Trainee competent in performing FC, or a consultant, provided supervision during each procedure.

### Outcome measures

FC was deconstructed into five key components for the purpose of assessment; these were defined prior to starting the project. The components were based on FC steps recommended by the Intercollegiate Surgical Curriculum Programme (ISCP) and the British Association of Urology Nurses for training and assessment (Table 1) [4–6].

**Table 1:** Five components of endoscopic assessment

	Area of assessment	Steps included	UFR	AFR
1.	Atraumatic passage of the cystoscope into the Bladder	Insertion of instillagel Insertion under direct vision Insertion of cystoscope into the bladder	3 %	1 %
2.	Examination of body and dome of bladder	Examine the dome, right and left lateral wall and posterior wall	3 %	1 %
3.	Examination of trigone and ureteric orifice		15 %	5 %
4.	Examination of bladder neck	Performed by inverting the cystoscope	3 %	1 %
5.	Performance of the full procedure		15 %	5 %

*UFR* unacceptable failure rate, *AFR* acceptable failure rate

CUSUM analysis requires an acceptable and unacceptable failure rate to define success and competence. Consultant Urologists in the South West of England were emailed a link to an online questionnaire describing the study. In the questionnaire consultants were asked to allocate acceptable failure rates for each flexible cystoscopy step. Of 65 invited to participate, 13 consultants completed the questionnaire. Each step has a different acceptable and unacceptable failure rate to take into account more challenging steps of the procedure (Table 1).

### Data analysis

The Type I and Type II error rates for CUSUM analysis were set at 0.10, as a standard. [7, 8] The acceptable and unacceptable failure rates varied with each step, however, were critical for the derivation of a CUSUM score. The CUSUM score (S) was calculated for each step (Additional file 1). For each success, a decrement of (S) is applied and each failure, an increase of (1 – S) is applied. The total score is cumulative and the total score is plotted as a continual plotted line.

Horizontal control lines are plotted at regular intervals on the y-axis. The spacing between control lines is calculated using a standardised equation (Additional file 1).

### Graph interpretation

The CUSUM value is plotted on the y-axis against the number of procedures on the x-axis. The CUSUM plotted line is a running sum of increments (1- S) and decrements (S).

Therefore, if the plotted line crosses the control line in an upward trend, performance is deemed unacceptable. If the control line is crossed in a downward trend then performance is deemed acceptable. If performance is maintained between two control lines then acceptable performance is being maintained. Competence is declared when 2 consecutive control lines are crossed in a downward fashion [9, 10].

## Results

Four hundred nineteen FCs were performed under local anaesthesia during the study period in 251 Males and 168 Females, median age 70 years (range 16–96). Indications for FC are detailed in Table 2.

**Table 2:** Indications for flexible cystoscopy

Indication	Number of patients (*n* = 417)
Haematuria	226 (54 %)
Surveillance for Bladder Cancer	149 (36 %)
Recurrent Urinary Tract Infections	16 (4 %)
To identify a stricture	9 (2 %)
Lower Urinary Tract Pain	6 (1 %)
Bladder stones	4 (1 %)
Neobladder surveillance	3 (1 %)
Lower Urinary Tract Symptoms	2 (<1 %)
Suspected fistula	1 (<1 %)
Surveillance follow Ureteric Cancer	1 (<1 %)

Each of the five steps has been evaluated individually and is presented in the order FC is performed. For each step, a number of procedures were excluded due to pathology preventing complete examination. The pathologies excluded were: urethral stricture, gross haematuria, obscuring malignancy and bladder stones.

1. Atraumatic passage of cystoscope via urethra
  Figure 1 shows the CUSUM score plotted against the number of procedures. It can be seen that the unacceptable control line [crossing the horizontal control line on the y-axis in an upwards fashion (positive gradient)] is first crossed at attempt 19 and again at attempt 47. During the first 51 attempts (Maximal point) there was a failure rate of 12 %. Performance begins to improve (no further unacceptable control lines crossed) and acceptable performance can be first concluded following attempt 117 when the acceptable control line is crossed [crossing the horizontal grid line from above to below (negative gradient)] [8]. Performance continues to improve crossing two further control lines following attempt 224 and 332. This component successfully completed by 117 procedures and competence following 224 procedures.

**Fig. 1:**
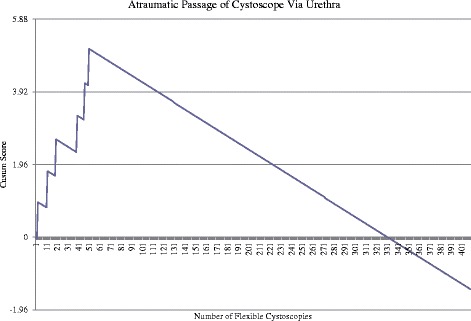
Cumulative Sum (CUSUM) score for atraumatic passage of cystoscope via urethra (*n* = 409). Acceptable failure rate at 0.01 and unacceptable failure rate at 0.03. Type I and II error rate 0.1. Each horizontal line on the y-axis represents a control line (h = 1.96). Filled arrow identifies when competence is achieved
2. Assessment of Body and Dome of Bladder
  Figure 2 shows the unacceptable control line (horizontal line crossed in an upwards fashion) is first crossed following attempt 3. The maximal point is achieved following 4 procedures and no further unacceptable control lines are crossed. Acceptable performance can be concluded following attempt 59 when the acceptable control line is crossed (crossing the horizontal line in a downwards fashion). No further failures occur and performance continues to improve, crossing two further control lines following attempt 221 and 330.

**Fig. 2:**
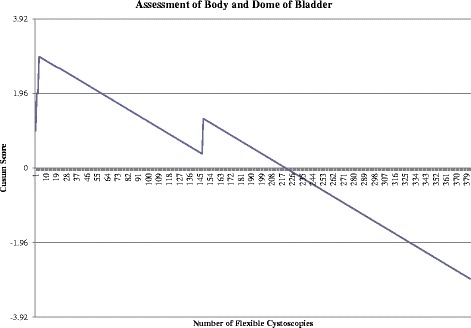
Cumulative Sum (CUSUM) for assessment of body and dome of bladder (*n* = 384). Acceptable failure rate at 0.01 and unacceptable failure rate at 0.03. Type I and II error rate 0.1. Each horizontal line on the y-axis represents a control line (h = 1.96)
  This component successfully completed by 59 procedures and competence following 221 procedures.
3. Identification of Trigone and Ureteric Orifices
  Figure 3 shows the unacceptable control line (horizontal line crossed in an upwards fashion) is first crossed following the 3^rd^ attempt with a further 15 unacceptable control lines continually crossed. Although the first acceptable control line is crossed following the 74^th^ attempt, the overall trend continues upwards until attempt 136. From attempt 136 to 257 satisfactory performance is evident as no further unacceptable control lines are crossed. Three acceptable control lines are crossed following 258^th^ to 301^st^ procedure, although the unacceptable control line is crossed following 302^nd^ procedure. No further unacceptable control lines are crossed in the final 81 procedures. Competence achieved following 279 procedures.

**Fig. 3:**
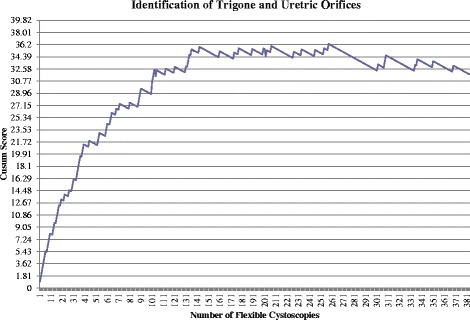
Cumulative Sum (CUSUM) for identification of trigone and ureteric orifices (*n* = 383). Acceptable failure rate set at 0.05 and unacceptable failure rate at 0.15. Type I and II error rates 0.10. Each horizontal line on the y-axis represents a control line (h = 1.81)
  Due to the large number of acceptable and unacceptable control lines being crossed during CUSUM score, the average failure rate was calculated with the number of procedures being divided into thirds. The initial failure rate for the first 128 attempts was 34 %. Between the 128^th^ and 254^th^ procedures the failure rate improved to 9 % then continued to improve further with a failure rate of 5 % between the 254^th^ and 383^rd^ procedure.
4. Examination of the bladder neck
  Figure 4 shows the unacceptable control line (horizontal line crossed in an upwards fashion) is first crossed following attempt 2 and again following attempt 4, 11 and 23. The maximal point is reached following the 23^rd^ procedure. Acceptable performance can be concluded following the 65^th^ procedure when the acceptable control line is crossed (crossing the horizontal line in a downwards fashion). However, failure following the 103^rd^ procedure leads to an unacceptable control line being crossed. No further unacceptable control lines are crossed following this procedure, with the acceptable control line being crossed following the 121^st^, 229^th^ and 338^th^ procedure.

**Fig. 4:**
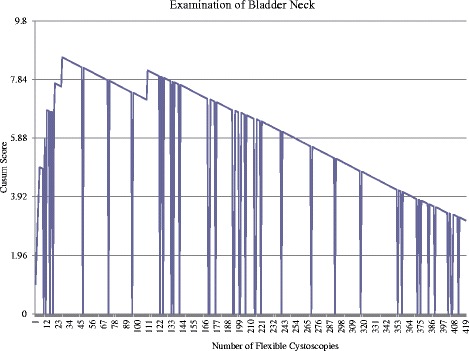
Cumulative Sum (CUSUM) for examination of the bladder neck (*n* = 383). Acceptable failure rate set at 0.01 and unacceptable failure rate set at 0.03. Type I and II error rate 0.1. Each horizontal line on the y-axis represents a control line (h = 1.96)
  This component successfully completed by 103 procedures and competence following 229 procedures.
5. Performance of full procedure
  Figure 5 shows the unacceptable control line first crossed following the 2^nd^ attempt with a further 19 unacceptable control lines continually crossed. Acceptable performance is first achieved when the control line is crossed following the 118^th^ procedure. However, acceptable performance is not maintained with multiple unacceptable control lines crossed. The maximal point was reached following 257^th^ procedure with a sustained period of acceptable performance following this, crossing 2 consequence acceptable control lines following the 286^th^ procedure. Competence achieved following 286 procedures.

**Fig. 5:**
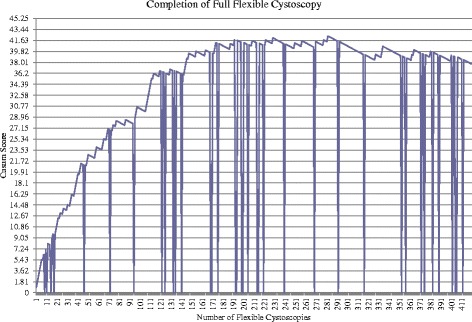
Cumulative Sum (CUSUM) score for completion of full flexible cystoscopy (*n* = 383). Acceptable failure rate set at 0.05 and unacceptable failure rate at 0.15. Type I and II error rates 0.10. Each horizontal line on the y-axis represents a control line (h = 1.81)
  Due to the large number of acceptable and unacceptable control lines being crossed, the average failure rate was calculated with the number of procedures being divided into thirds. The failure rate of the first 128 procedures was 38 %. The failure rate between 128^th^ and 256^th^ procedure was 13 %. Improvement continued with a failure rate of 6 % between 257^th^ and 383^rd^ procedure.

## Discussion

This study describes the learning curve to competency in FC of a trainee with no previous experience in endoscopy under supervision. In an era of quality assurance and credentialing the results provide further evidence that CUSUM analysis is an objective technique, which can be used to evaluate progression to competence.

The prospective data in the current study demonstrate that CUSUM is a relatively simple and sensitive method to apply practically to self assessment in surgical training. CUSUM was able to highlight areas for improvement, guiding further training in addition to defining competence. In this study, the trainee took longer to achieve, and maintain, competency than suggested indicative numbers [1].

The utility of CUSUM was that it was able to define the specific aspects of the procedure which the trainee found most difficult. For three out of five of the components, acceptable performance was maintained following the 122nd procedure. Competence in examination of the ureteric orifices and trigone was a FC step which took substantially longer to acquire, being achieved by the 280th procedure. CUSUM highlighted this as an area for targeted tuition in this trainee.

Even if it is assumed that performing a minimum number of procedures will result in competence the number of FCs required to attain competence has never been validated. BAUS recommended a minimum of 60 procedures in 2000 [1]. In 2014 the Speciality Advisory committee in Urology (SAC) stipulated that the indicative number of FCs which must be performed for the award of a Certificate of Completed Training (CCT) in Urology should be 300. Prior to this, a review of logbook data from trainees applying for CCT between 2010 and 2012 revealed that only 42 % had recorded flexible cystoscopy activity [11]. This wide range of recommendations and trainee activity highlights the need for an alternative method, such as CUSUM, to be introduced as a more robust modality for determining competence.

CUSUM analysis has not previously been used to assess skill acquisition in FC. Studies, using virtual reality simulators to assess skill acquisition for FC, have developed a five-point Global Rating Score (GRS). Although this has been of value in evaluating technical and nontechnical skills, it may be limited by inter assessor variability [12, 13]. Such variability does not occur with CUSUM analysis due to each defined task having a binary outcome. In addition CUSUM has the advantage of being suitable for both self-assessment and supervisor assessment. As a result, CUSUM analysis has the potential to be incorporated into the trainee’s curriculum with the plotted graphs providing readily visualised, accurate, comparable evidence of progression and competence rather than the current implicit logbook approach.

While ideally CUSUM could be used as a tool for assessing skill acquisition, and its maintenance, in trainees and consultants a significant issue is that the statistical calculations are detailed and time intensive. This may be one of the reasons why, to date, CUSUM has not been widely adopted. A possible development would be creation of software to facilitate the data entry and analysis.

The present study has limitations; CUSUM was demonstrated to be an objective technique however the authors acknowledge that only one trainee’s performance was assessed. Maguire et al. used CUSUM analysis for the evaluation of a group of trainees performing retropubic mid-urethral sling procedures [14]. In keeping with that study, our study found the number of procedures required to acquire, and maintain, competence in performing the procedure was significantly more than expected [14]. Furthermore, Maguire at al. identified considerable variability in the number of procedures each trainee needed to achieve competence [14]. For CUSUM to be used for FC assessment routinely its applicability would have to be evaluated in a larger trainee cohort where similar inter-trainee variability is likely be identified. The very least that such a study would achieve would be a more accurate estimate of the range of indicative numbers which a trainee requires to achieve, and maintain, competence.

A key element in CUSUM analysis is the determination of the acceptable and unacceptable failure rates of the FC procedure. Another possible limitation of this study is that these rates were based on a relatively small sample of thirteen consultants. Despite the sample being representative of the centres involved in training in the South West of England it would be desirable to increase this sample size in future studies.

Patient experience and complication rates are important outcome measures, which are integral to true competence. Currently these measures are not incorporated into UK urology trainees’ assessments. These factors were not assessed in this study as part of the CUSUM analysis because the focus was on FC performance. It would be appropriate in future studies to incorporate patient experience into CUSUM and a parallel audit to accurately capture complications.

## Conclusion

This study demonstrates the successful use of CUSUM analysis in the assessment of surgical competence for FC. The method is one, which could be used to assess, and monitor competence, in surgical trainees, however, validation of the process, using a larger trainee cohort, is required.

## Abbreviations

BAUS, British Association of Urological Surgeons; CCT, certificate of completed training; CUSUM, cumulative sum; FC, flexible cystoscopy; GRS, global rating score; ISCP, Intercollegiate Surgical Curriculum Programme; MRC, medical research council; NHS, National Health Service; SAC, Surgical Advisory Committee

## Additional files

Additional file 1: CUSUM Equation. Additional file 1 is the mathematical equation used to calculate the value to increase or decrease the CUSUM score along with the equation used to establish the interval between control lines for each graph. (PDF 56 kb) Additional file 2: NHS health research authority approval. Additional file 2 is the outcome of the online assessment by the NHS health research authority and the MRC of the study design. (PDF 133 kb)
