# Erratum to: Defining competency in flexible cystoscopy: a novel approach using cumulative Sum analysis

**DOI:** 10.1186/s12894-016-0175-1

**Published:** 2016-09-12

**Authors:** Kenneth R. MacKenzie, Jonathan Aning

**Affiliations:** Department of Urology, The Newcastle upon Tyne Hospitals NHS Foundation Trust, Freeman Hospital, Newcastle-Upon-Tyne, NE7 7DN UK

## Erratum

After publication of this work [1] it was noticed that the incorrect versions of Figs. 4 and 5 were used.

**Fig. 4:**
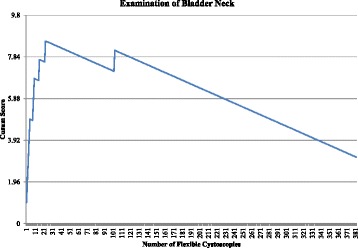
Cumulative Sum (CUSUM) for examination of the bladder neck (*n* = 383). Acceptable failure rate set at 0.01 and unacceptable failure rate set at 0.03. Type I and II error rate 0.1. Each horizontal line on the y-axis represents a control line (h = 1.96)]

**Fig. 5:**
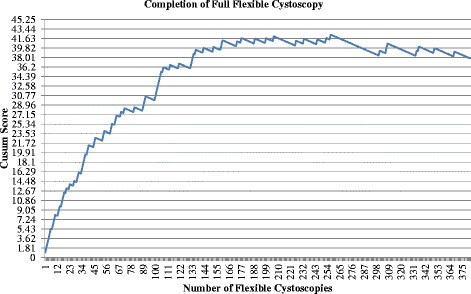
Cumulative Sum (CUSUM) score for completion of full flexible cystoscopy (*n* = 383). Acceptable failure rate set at 0.05 and unacceptable failure rate at 0.15. Type I and II error rates 0.10. Each horizontal line on the y-axis represents a control line (h = 1.81)]

Please see below the correct versions of Figs. 4 and 5.

We apologise for these errors.
