# Supplementary material for: Defining competency in flexible cystoscopy: a novel approach using cumulative Sum analysis
Source: BMC Urol. 2016 Jun 13;16:31. doi: 10.1186/s12894-016-0143-9 (PMC4907277; doi:10.1186/s12894-016-0143-9)
Supplement: Additional file 1: — CUSUM Equation. Additional file 1 is the mathematical equation used to calculate the value to increase or decrease the CUSUM score along with the equation used to establish the interval between control lines for each graph. (PDF 56 kb) [file 12894_2016_143_MOESM1_ESM.pdf]

Appendix 1:

For each area of assessment, (1-5), the CUSUM formula was applied. The intermediate variables a, b, P, Q and S were used as per Bolin and Colson [8]. Type I error rate (α) and the Type II error rate (β) were set at 0.10, as standard for CUSUM analysis [8].

$a=\ln[(1-\beta)/\alpha]$ $b=\ln[(1-\alpha)/\beta]$

The acceptable failure rate (p0) and unacceptable failure rate (p1) were set for each step of assessment as per table 1.

$P=\ln(p1/p0)$ $Q=\ln[(1-p0)/(1-p1)]$

Therefore, the decrement for each success on the CUSUM plot is:

$S=Q/(P+Q)$

The increment for each failure is (1-S)

The spacing between the unacceptable control lines (h0) and acceptable control lines (h1) are determined by:

$h0= b/(P+Q)$ $h1= a/(P+Q)$
